# Identification of emergent *bla*_*CMY-2*_-carrying *Proteus mirabilis* lineages by whole-genome sequencing

**DOI:** 10.1016/j.nmni.2015.11.012

**Published:** 2015-11-27

**Authors:** M. Mac Aogáin, T.R. Rogers, B. Crowley

**Affiliations:** 1) Department of Clinical Microbiology, Sir Patrick Dun Translational Research Laboratory, School of Medicine, Trinity College Dublin, Dublin, Ireland; 2) Department of Microbiology, St James's Hospital, Dublin, Ireland

**Keywords:** *bla*_*CMY-2*_, genomics, ICE, PGI-1, *Proteus mirabilis*

## Abstract

Whole-genome sequencing of 24 *Proteus mirabilis* isolates revealed the clonal expansion of two cefoxitin-resistant strains among patients with community-onset infection. These strains harboured *bla*_*CMY-2*_ within a chromosomally located integrative and conjugative element and exhibited multidrug resistance phenotypes. A predominant strain, identified in 18 patients, also harboured the PGI-1 genomic island and associated resistance genes, accounting for its broader antibiotic resistance profile. The identification of these novel multidrug-resistant strains among community-onset infections suggests that they are endemic to this region and represent emergent *P. mirabilis* lineages of clinical significance.

## Introduction

*Proteus* *mirabilis* is a significant cause of urinary tract infections and is a leading cause of catheter-associated urinary tract infections [1]. Treatment is complicated by the acquisition of antibiotic resistance genes affording *P. mirabilis* a selective advantage during therapy. Horizontal acquisition of AmpC-type β-lactamase genes has been an important driver of resistance in Europe and has been associated with the clonal expansion of resistant strains [2], [3]. Since the publication of the *P. mirabilis* HI4320 reference genome, additional genome sequences have become available providing a framework for genomic epidemiology in this species [4], [5], [6], [7], [8], [9]. Here, we applied whole-genome sequencing to investigate the genomic epidemiology of emergent cefoxitin-resistant *P. mirabilis* isolates causing community-onset infections in Ireland.

## Methods

Clinical *P*. *mirabilis* isolates, recovered by the Microbiology Department of St James's Hospital (Dublin, Ireland), were subjected to antimicrobial sensitivity testing on a Vitek 2 system (bioMérieux, Marcy l’Étoile, France). Infections with onset in the community were categorized as either community acquired, when onset of illness occurred outside a healthcare facility with no reported discharge from a healthcare facility within the previous 12 weeks, or healthcare associated, when onset of illness occurred within 4 weeks of discharge from a healthcare facility. Whole-genome sequencing of *P. mirabilis* isolates was performed on an Illumina MiSeq platform at the TrinSeq sequencing facility (Trinity College Dublin, Ireland). Sequencing reads were aligned to the *P. mirabilis* HI4320 genome (AM942759) using the Burrows-Wheeler short-read aligner, while *de novo* assembly was performed using the NSilico Simplicity pipeline (Simplicity v1.2) [2], [10]. Sequence data for the *P. mirabilis* integrative and conjugative element ICEPmiJpn1 (AB525688.1) and Proteus Genomic Island-1 (KJ411925) were also used as reference sequences for read mapping and contig alignment. Single-nucleotide variants (SNVs) were resolved using SAMtools [11]. Phylogenetic trees were generated by neighbour-joining (BIONJ) using PhyML and visualized with iTOL [12], [13]. Raw short-read data have been deposited at the European Nucleotide Archive under study accession number PRJEB7631. Draft assemblies of representative cefoxitin-resistant strains PM655 and PM593 have been deposited at DDBJ/EMBL/GenBank under accession numbers JSUO00000000 and JSUP00000000, respectively.

## Results

**Fig. 1:**
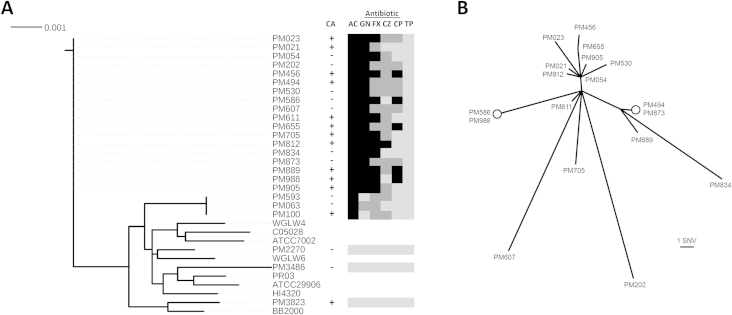
Phylogenetic comparison of Irish *Proteus mirabilis* isolates with previously sequenced *P. mirabilis* strains. (A) A neighbour-joining tree was generated on the basis of concatenated sequences of seven conserved genes present in all strains (*adk*; *PMI0375*, *fumC*; *PMI0891*, *gyrB*; *PMI1296*, *icd*; *PMI2184*, *mdh*; *PMI1732*, *purA*; *PMI3370* and *recA*; *PMI3400*). Sequences from 24 *P. mirabilis* isolates from this study and eight representative *P. mirabilis* whole-genome data sets; C05028 (ANBT00000000), WGLW4 (AMGU00000000), HI4320 (AM942759), ATCC29906 (ACLE01000000), ATCC7002 (JOVJ00000000), PR03 (AORN00000000), WGLW6 (AMGT00000000) and BB2000 (CP004022) were compared. Black bar indicates average nucleotide substitutions per site across the seven genes analysed (9 kb). To the right of the tree, under label CA, plus and minus symbols denote either community-acquired or hospital-associated infections, respectively, while subsequent columns ‘AC,’ ‘GN,’ ‘FX,’ ‘CZ,’ ‘CP’ and ‘TP’ indicate the resistance profile of each isolate to amoxicillin–clavulanic acid, gentamicin, cefoxitin, ceftazidime, ciprofloxacin and piperacillin–tazobactam, respectively. Colour indicates resistance level: black, full resistance; dark grey, intermediate resistance; light grey, susceptible. (B) Unrooted neighbour-joining tree of the observed 18-strain clonal cluster based on whole-genome comparisons across 3,207,626 called sites relative to the *P. mirabilis* HI4320 genome. Circles indicate genetically indistinguishable strains. Bar below the tree indicates length corresponding to one single-nucleotide variant.

Between December 2012 and November 2013, 33% of *P*. *mirabilis* isolates (*n* = 79) recovered by the Microbiology Department of St James's Hospital were found to exhibit resistance to cefoxitin (minimum inhibitory concentration ≥16 mg/L). Twenty-one cefoxitin-resistant isolates, confirmed as being from community-onset infections, were further investigated by whole-genome sequencing. Cefoxitin-resistant isolates had multidrug-resistance (MDR) phenotypes, exhibiting coresistance to other antibiotics including amoxicillin–clavulanic acid (100%), gentamicin (91%), ceftazidime (76%) and ciprofloxacin (52%) but remained sensitive to piperacillin–tazobactam, with the three control isolates exhibiting susceptibility to all antibiotics tested (Fig. 1(A)). No epidemiologic links between cases could be established on analysis of patient records, suggesting that potential strain bias due to patient-to-patient transmission was minimal.

Whole-genome sequence data indicated that our resistant isolates exhibited significant genetic divergence from available *P. mirabilis* genome sequences present in the Refseq database and included a large cluster of 18 genetically related isolates (Fig. 1(A)). Genome-wide analysis of the 18-isolate cluster (cluster I) revealed isolates to be highly clonal, exhibiting a genetic divergence of between 0–30 SNVs (Fig. 1(B)). Similarly, within the smaller isolate cluster (cluster II, *n* = 3), isolates PM100 and PM593 were genetically indistinguishable, whereas PM063 diverged by 20 SNVs (isolates in cluster I diverged from those of cluster II by more than 19,000 SNVs).

**Fig. 2:**
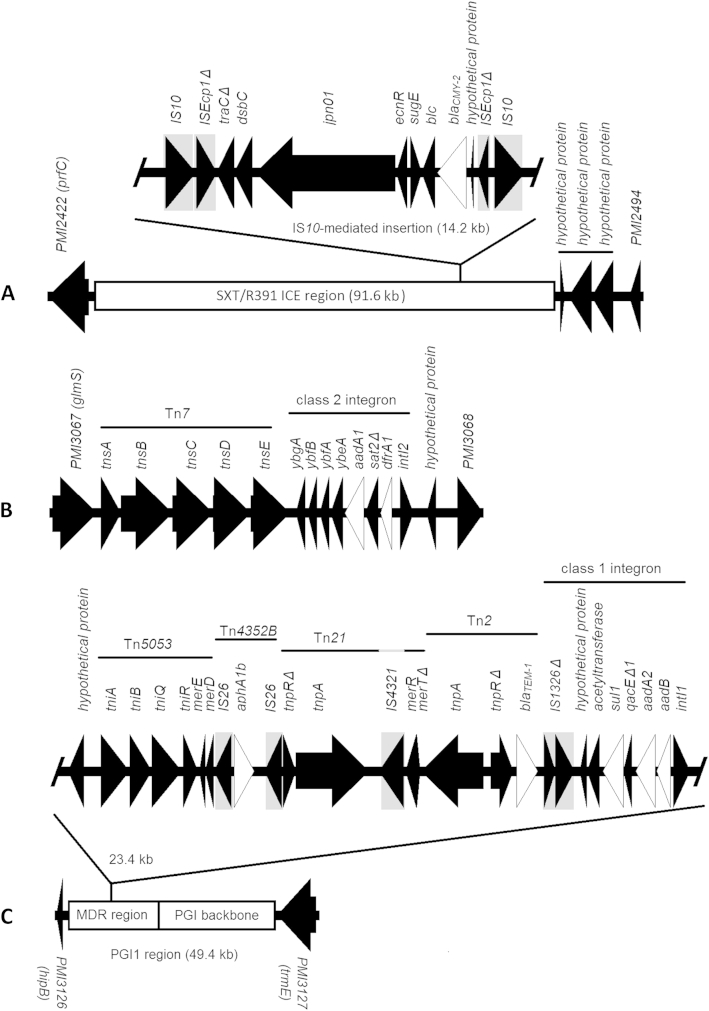
Observed mobile genetic elements associated with chromosomally integrated resistance genes among *Proteus mirabilis* strains identified in this study. Genetic environs (black) of detected resistance genes (white) among resistant *P. mirabilis* isolates are illustrated. Identified insertion sequences are highlighted in grey boxes. (A) Both strains harboured the described integrative and conjugative element (ICE) element ICE*Pmi*Jpn1, which was integrated between chromosomal genes *PMI2422* (*prfC*) and *PMI2949*. The *bla*_*CMY-2*_ gene was present within an ICE-embedded composite transposon flanked by IS*10* elements, as observed in ICE*Pmi*Jpn1 (AB525688.1). The ICE observed in cluster I isolates also included three additional genes encoding hypothetical proteins (underlined), which are absent from previously described ICE elements in *P. mirabilis*. (B) Cluster I isolates harboured the *aadA1* and *dfrA1* genes associated within a Tn7-associated class 2 integron, chromosomally located between *PMI3067* (*glmS*) and *PMI3068*. (C) PGI-1 genomic island was identified among cluster I isolates, chromosomally integrated between *PMI3127* (*hipB*) and *PMI3127* (*trmE*). Resistance genes identified within the PGI-1 region included *aphA1b, bla*_*TEM-1*_*, sul1, aadA2* and *aadB*, which were embedded among a mosaic of mobile genetic elements in the PGI-1 MDR region.

An R391/SXT-family integrative and conjugative element (ICE), harbouring the *bla*_*CMY-2*_ AmpC-family β-lactamase gene, was identified in all cefoxitin-resistant strains (both in cluster I and cluster II). This ICE shared high sequence identity to ICE*Pmi*Jpn1 (AB525688.1) and similarly contained the *bla*_*CMY-2*_ gene within a composite transposon insertion [14]. The identified ICE region was integrated at the same chromosomal location in both strains (Fig. 2(A)). Cluster I isolates also harboured additional chromosomally integrated resistance genes; a Tn*7*-associated class 1 integron was present upstream of *PMI3067* (*glmS*) carrying the *aadA1* and *dfrA1* resistance genes (Fig. 2(B)), while the Proteus Genomic Island 1 (PGI-1) was also identified [15]. PG1-1 harboured the resistance genes *aadB*, *aad2*, *aphA1b*, *bla*_*TEM*-*1*_ and *sul1* associated with diverse mobile elements within its MDR region while lacking the “right-end” of the PGI-1 MDR region as well as the IS*26*-mediated recombination event originally described among French *P. mirabilis* isolates (Fig. 2(C)) [15]. The three antibiotic-susceptible isolates investigated lacked the R391/SXT-family ICE, class 2 integron-borne *aadA1* and *dfrA1*, and PGI-1. Although we failed to identify mutations within the quinolone-resistance-determining regions of *gyrA, gyrB, parC* or *parE* among ciprofloxacin-resistant isolates, loss-of-function mutations in transcriptional regulators of efflux *acrR* (frameshift: c.90_93dup) and *soxR* (stop gain; p.Gln147Stop) were identified in four of five resistant isolates exhibiting ciprofloxacin minimum inhibitory concentrations of >4 mg/L. These mutations were absent from susceptible (*n* = 13) or intermediately resistant (*n* = 6) isolates.

## Discussion

*Proteus mirabilis* exhibits a clonal population structure whereby the emergence of pathogenic strains is often observed in association with the acquisition of exogenous antibiotic resistance genes [3], [14], [16]. Here the application of whole-genome sequencing allowed fine-structure comparative genomic analysis of locally emergent *P. mirabilis* strains identifying two novel clonal lineages among community-onset infections. While both strains harboured the ICE-located *bla*_*CMY*-*2*_, accounting for observed cephamycin resistance, cluster I strains were distinguished by the presence of a chromosomally located Tn*7*-associated class 1 integron and the PGI-1 genomic island. These gene mobilization platforms harboured several additional antibiotic resistance genes accounting for the broader resistance profile of the cluster I strain such as invariable gentamicin resistance, which was accounted for by the presence of class 1 integron-associated *aadB* in the PGI-1 MDR region (Fig. 2(C)). Thus, the acquisition of multiple resistance genes *via* distinct gene mobilization platforms has likely been a key contributor to the clonal expansion of this novel strain among community-onset infections. Worryingly, a number of cluster I isolates were also resistant to ciprofloxacin, further limiting therapeutic scope for treatment of community-acquired infection. While the mechanism of fluoroquinolone resistance in these strains awaits formal validation, mutational disruptions in *soxR* and *acrR* were noted. Similar mutations in *acrR* and *soxR* are known to contribute to fluoroquinolone resistance in other pathogenic members of the *Enterobacteriaceae* by altering expression of the AcrAB efflux system, while increased expression of *acrB* has been confirmed among fluoroquinolone-resistant *P. mirabilis* isolates [17], [18], [19]. The predominance of the MDR strains described here among apparently epidemiologically unrelated patients suggests that they represent *P. mirabilis* lineages endemic to this region, warranting further local surveillance. It will be interesting to assess whether these strains are localized to this region or have a broader global distribution. In the absence of a widely accepted and portable typing scheme in *P*. *mirabilis*, whole-genome sequencing provides an important tool in addressing such questions.
